# Mechanisms of platelet activation in cancer-associated thrombosis: a focus on myeloproliferative neoplasms

**DOI:** 10.3389/fcell.2023.1207395

**Published:** 2023-06-29

**Authors:** Roelof H. Bekendam, Katya Ravid

**Affiliations:** ^1^ Division of Hematology and Hematologic Malignancies, Beth Israel Deaconess Medical Center, Harvard Medical School, Boston, MA, United States; ^2^ Department of Medicine and Biochemistry, Whitaker Cardiovascular Institute, Boston University Chobanian & Avedisian School of Medicine, Boston, MA, United States

**Keywords:** myeloproliferative neoplasms, polycythemia vera, essential thrombocythemia, myelofibrosis, cancer associated thrombosis

## Abstract

Platelets are anucleate blood cells that play key roles in thrombosis and hemostasis. Platelets are also effector cells in malignancy and are known to home into the microenvironment of cancers. As such, these cells provide central links between the hemostatic system, inflammation and cancer progression. Activation of platelets by cancers has been postulated to contribute to metastasis and progression of local tumor invasion. Similarly, cancer-activated platelets can increase the risk of development of both arterial and venous thrombosis; a major contributor to cancer-associated morbidity. Platelet granules secretion within the tumor environment or the plasma provide a rich source of potential biomarkers for prediction of thrombotic risk or tumor progression. In the case of myeloproliferative neoplasms (MPNs), which are characterized by clonal expansion of myeloid precursors and abnormal function and number of erythrocytes, leukocytes and platelets, patients suffer from thrombotic and hemorrhagic complications. The mechanisms driving this are likely multifactorial but remain poorly understood. Several mouse models developed to recapitulate MPN phenotype with one of the driving mutations, in JAK2 (JAK2V617F) or in calreticulin (CALR) or myeloproliferative leukemia virus oncogene receptor (MPL), have been studied for their thrombotic phenotype. Variability and discrepancies were identified within different disease models of MPN, emphasizing the complexity of increased risk of clotting and bleeding in these pathologies. Here, we review recent literature on the role of platelets in cancer-associated arterial and venous thrombosis and use MPN as case study to illustrate recent advances in experimental models of thrombosis in a malignant phenotype. We address major mechanisms of tumor-platelet communication leading to thrombosis and focus on the role of altered platelets in promoting thrombosis in MPN experimental models and patients with MPN. Recent identification of platelet-derived biomarkers of MPN-associated thrombosis is also reviewed, with potential therapeutic implications.

## Overview

Individuals with cancer are at increased risk of both arterial and venous thromboembolism (Khorana et al., 2007; Navi et al., 2017). Cancer-associated thrombosis is the second most common cause of death in patients with cancer, after death by cancer progression. Further, thrombocytosis has been associated with poorer survival in patients with several cancers, such as ovarian cancer (Stone et al., 2012). Platelet interaction with tumor cells is necessary for successful hematogenous metastasis (Anvari et al., 2021). Circulating tumor cells use a plethora of mechanisms to adhere to platelets within the vasculature, which allows for evasion of immune surveillance (Lin et al., 2021). Platelets are thought to coat tumor cells, which leads to diminished recognition by natural killer cells (Ward et al., 2021). Tumor cells interact with platelets through various mechanisms (Roweth and Battinelli, 2021). Soluble mediators enhance platelet-tumor interactions, increase vascular permeability to enhance transendothelial tumor migration, tumor proliferation and further platelet-induced chemotaxis of protumorigenic monocyte and mother myeloid cell invasion. Perhaps almost as an unwarranted side-effect, these soluble mediators promote prothrombic platelet and coagulation cascade activation, increasing the risk of cancer associated thrombosis (CAT). This review screens major mediators of platelet-cancer cells interactions, with focus on thrombosis in states of myeloproliferative neoplasms (MPN), and accordingly, possible biomarkers of MPN-associated thrombosis.

### Tumor-platelet communication leading to thrombosis through tumor-expressed mediators

#### Extracellular vesicles and the role of thrombin

Tumor cells have the capacity to increase thrombin generation through direct and indirect mechanisms. Different cancer cells express tissue factor (TF) on their membranes. TF can activate the coagulation cascade through a series of serine protease activation reactions leading to the conversion of prothrombin to thrombin. Thrombin has various substrates; it can convert fibrinogen to fibrin and cleave the protease activating receptor-1 (PAR-1) on platelets to induce platelet activation and secretion. The initial observations that tumor cells could induce platelet activation relied on co-culture systems where after addition of a direct thrombin inhibitor, tumor cell-induced platelet aggregation (TCIPA) was inhibited (Heinmoller et al., 1996). Extracellular vesicles (EVs) released by cancer cells provide another procoagulant surface that can lead to direct thrombin generation in patients with cancer. Most efforts have been directed towards detecting TF-expressing EVs, which have been correlated with thrombin generation in several cancers [reviewed by Gardiner et al. (2015)]. Targeting the coagulation cascade, ultimately decreasing thrombin generation, has been the hallmark of treatment of CAT. Low molecular weight heparins (LMWHs), increasing deactivation of thrombin by activating antithrombin, were found to be more effective than vitamin K antagonists in a pivotal trial studying patients with cancer associated thrombosis (Lee et al., 2003). More recently, direct anticoagulants, such as targeting FXa, were shown to be safe and effective in most patients with CAT (Raskob et al., 2018; Agnelli et al., 2020).

Subsets of EVs were shown to carry polyphosphates, while polyphosphates were reported to activate the intrinsic pathway of the coagulation cascade, ultimately leading to increased thrombin formation. (Morrissey and Smith, 2015) Specifically, samples from patients with colon cancer, prostate cancer and pancreatic cancer activate the coagulation cascade in a FXII-dependent fashion (Nickel et al., 2015; Shim et al., 2021). Similarly, incubation of plasma with promyelocytic leukemia cells leads to FXII activation (Nickel et al., 2015).

#### Platelet dysfunction via ADP and its receptors

Tumors are also able to secrete adenosine diphosphate (ADP) (Bastida et al., 1986; Boukerche et al., 1994). ADP can interact with its receptors on platelets, P2Y1 and P2Y12. Binding of ADP to these receptors leads to platelet degranulation. P2Y12 inhibition by depleting ADP with apyrase or directly with 2-methylthio-AMP reduced platelet aggregation otherwise induced by a cell line of breast carcinoma (Alonso-Escolano et al., 2004). Similarly, in a mouse model of ovarian cancer, pharmacological inhibition of P2Y12, or knockout of P2Y12 resulted in a significant reduction of tumor size (Cho et al., 2017). In contrast, P2Y1 knockout did not show a difference in tumor growth in mouse models of metastatic pancreatic cancer (Palacios-Acedo et al., 2021). Pharmacological P2Y12 inhibition was shown to be more effective at dissolving spontaneous endogenous thrombi in the same study. More recently, in a mouse model of liver cancer, platelets were shown to inhibit liver cancer growth, but P2Y12 inhibition enhanced tumor growth (Ma et al., 2022). Overall, some, but not all reported studies point to secreted ADP and platelet P2Y12 as important mediators of tumor growth in preclinical models, and as potential therapeutic targets for thrombosis in neoplastic conditions. While pharmacological P2Y12 inhibition has been widely used in arterial thrombosis prevention, stroke management and prevention of in-stent thrombosis [reviewed by Capodanno and Angiolillo (Capodanno and Angiolillo, 2023)], its role in CAT has not been evaluated in randomized control trials. Patients actively undergoing treatment with anti-neoplastic agents can be at risk for severe thrombocytopenia, complicating the use of functional anti-platelet agents in this population. Observational data in patients receiving P2Y12 agents showed conflicting data concerning cancer development. Two large trials comparing P2Y12 inhibition to aspirin did not find a change in cancer incidence between the groups (Hicks et al., 2015; Leader et al., 2017). The TRITON-TIMI 28 trial compared two P2Y12 inhibitors (prasugrel and clopidogrel) in the setting of acute myocardial infarction and showed a significant detection rate of colonic cancer in the prasugrel arm (Wallentin et al., 2009). Similarly, in the DAPT and PEGASUS trials, clopidogrel, prasugrel and ticagrelor accelerated cancer-related deaths. Overall, these results need to be interpreted carefully as no untreated comparator arm was involved, and the studies were not powered to detect differences for cancer-related events. Indeed, the interplay between different parts of the coagulation cascade, platelets and the tendency to develop cancer-associated thrombosis remains to be further evaluated. For example, mouse models expressing an orthotopic pancreatic cancer high in TF did not have diminished thrombus formation after FXI silencing (Hisada et al., 2021). These observations emphasize the importance of individualized approaches regarding type of anticoagulants, anti-platelet agents or agents targeting interactions between different players within the vasculature.

#### Platelet-neutrophil interactions in cancer-associated thrombosis

Platelets are uniquely versatile and able to interact with a plethora of cells within the vasculature. They home to the micro-environment of tumors and are considered a part of it (reviewed by Schlesinger) (Schlesinger, 2018). Recently, it has been established that platelet-leukocyte interactions contribute significantly to thrombotic phenotypes observed in cancer patients (Sreeramkumar et al., 2014; Mauracher et al., 2018). Multiple groups have evaluated the role of neutrophil extracellular traps (NETs) in cancer-associated thrombosis (Demers et al., 2012; Thalin et al., 2019; Rosell et al., 2021). NETs are thought to be part of the innate immune system and have a primary role in engulfing microbes (Brinkmann et al., 2004). NETs are the result of neutrophil chromatin, histone and granule expulsion. However, in recent years it has become clear that NETs can also contribute to various pathological processes, including pathological clotting (Fuchs et al., 2010). Platelets of patients with cancer prime neutrophils to release more NETs (Olsson and Cedervall, 2016). Platelets can interact with neutrophils through various mechanisms to stimulate NETosis. For instance, in sepsis models using lipopolysaccharide (LPS) platelet-neutrophil interactions were found to significantly increased and were dependent on TLR-4 (Clark et al., 2007). LPS-primed platelets led to a significant increase in NETosis. Classical platelet agonists, including thrombin and ADP, have been shown to trigger the formation of NETs. This process is reliant on P-selectin glycoprotein ligand-1 (PSGL-1) (Etulain et al., 2015). The authors demonstrate, using a P-selectin knockout mouse model, that NETs cannot be formed in response to thrombin-activated P-selectin knockout platelets. The secretion of other granule content remains unchanged, indicating that the interaction between PSGL-1 and P-selectin is crucial for the initiation of NETosis.

NETosis further enhances platelet activation through several mechanisms. First, NETs provide a negative charged surface that can allow for the activation of factor XII to XIIa (Vu et al., 2016). Through a series of serine protease activation in the coagulation cascade, this ultimately leads to a further amplification of thrombin generation. Second, von Willebrand Factor (vWF) is able to attach to the exposed DNA, allowing for capture of platelets through adhesion (Fuchs et al., 2010). These two mechanisms allow for increased platelet activation at the site of NETosis. Thirdly, release of histones from neutrophils during NETosis can directly activate platelets in a TLR-2 and TLR-4 dependent manner (Semeraro et al., 2011). Specifically, histone H4 on NETs has been implicated in release of platelet polyphosphates. The interplay between platelet derived polyphosphates and NETosis is part of a positive feedback loop in which polyphosphates and NETs both amplify coagulation through a FXII-dependent mechanism. Platelet derived polyphosphates can induce NETosis *in vitro* and inhibition with monoclonal antibodies targeting polyphosphates diminishes NET induced intravascular thrombin generation (McDonald et al., 2017).

Strikingly, it was observed that patients with cancer and leukocytosis are more prone to develop VTE (Blix et al., 2013). Preclinical models of several cancers, including breast, pancreatic and lung, led to tumor mediated NET formation (Demers et al., 2012; Rayes et al., 2019). Similarly, it was shown that mice bearing these tumors had neutrophilia and increased histone levels (Demers et al., 2016). Pretreatment with DNAse I prior to induction of venous thrombosis by (inferior vena cava) IVC ligation resulted in decreased thrombus weight in tumor-bearing mice, but not in control mice (Hisada et al., 2020). This indicates that NETosis represents an important mechanism by which cancers induce thrombosis. Clinical trials with DNAse in CAT are currently underway. However, extrapolating from the presented evidence, disrupting the platelet-neutrophil interplay, may be an attractive target for inhibition of NETosis and subsequently VTE in CAT.

#### Podoplanin and its ligands

Podoplanin, a heavily glycosylated transmembrane glycoprotein, is often upregulated in several types of cancer, including squamous cell carcinomas, glioblastoma and mesotheliomas (Ordonez, 2014). Next to its expression in malignant cells, podoplanin has been observed in alveolar cells in the lung, podocytes in the kidney and within the lymphatic system. Its physiological role, however, remains largely unknown. In the mid-2000s, C-type lectin receptor (CLEC-2) had been found as a novel receptor on platelets that can be activated by the snake venom rhodocytin. Activation of the CLEC-2 receptor by rhodocytin leads to platelet aggregation and platelet activation in a Syk-dependent fashion (Suzuki-Inoue et al., 2006). In a follow up paper from the same group, it was elegantly described how podoplanin-induced platelet aggregation and activation was reminiscent of rhodocytin (Suzuki-Inoue et al., 2007). In this seminal paper it was shown that the target molecule of podoplanin on the platelet surface is CLEC-2. These observations have led to the study of the podoplanin-CLEC-2 axis in CAT. Mouse models using orthotopic podoplanin-bearing tumors or injection of podoplanin-tumors into mice led to the observations that both metastasis and pathological thrombosis is dependent on the podoplanin-CLEC-2 axis (Riedl et al., 2017; Shirai et al., 2017). Depletion of CLEC-2 in mice bearing a podoplanin expressing melanoma led to decreased thrombus formation (Shirai et al., 2017). Interestingly, complete inducible or platelet-specific CLEC-2 knockout leads to diminished thrombus formation in a deep vein thrombosis (DVT) model of inferior vena cava stenosis in mice without cancer (Suzuki-Inoue et al., 2010; Riedl et al., 2017). Podoplanin becomes upregulated in the inflamed vessel wall, and it is postulated a similar mechanism may play a role in CAT as well. Clinical studies on patients with glioma found that podoplanin expression correlated with increased levels of D-dimer and an increased risk of VTE (Riedl et al., 2017). Metastasis of primary brain tumors is quite rare; therefore, the authors postulate that the increased risk of VTE may be due to circulating microvesicles bearing podoplanin. Targeting CLEC-2 may have advantages over targeting podoplanin in CAT. CLEC-2 is mainly expressed in megakaryocytes and platelets, whereas podoplanin is expressed both in healthy cells and is pathologically upregulated in several cancers. Efforts to target this axis have remained pre-clinical for now, but include small molecules, such as anti-CLEC2 antibodies and a mutant rhodocytin (Harbi et al., 2021). Further evaluation is necessary before the transition to clinical studies can be safely undertaken.

### Thrombosis in MPN

#### Overview

MPNs include polycythemia vera (PV), essential thrombocythemia (ET), and primary myelofibrosis (PMF), as observed in clinical and bone marrow morphologic findings (Vardiman et al., 2009). A number of mutations have been described in this group of diseases, including JAK2V617F hyper-activating mutation, which is detected in 90%–95% of PV, 55% of ET, and 65% of primary myelofibrosis (PMF) cases, and exon 9 mutations in the calreticulin gene *CALR* (James et al., 2005; Kralovics et al., 2005; Zhao et al., 2005; Tefferi and Vainchenker, 2011). Reported incidence of thrombosis ranges from 12% to 39% in PV and from 11% to 25% in ET, and the rate of major cardiovascular events in PMF is comparable with that reported in ET (Barbui et al., 2010). Data from the Swedish Cancer Register from 1980 to 2009 showed that in a cohort of 11,155 patients with MPNs and 44,620 matched healthy controls, the risk of arterial thrombosis was significantly 4.9-fold (4.8–5.0 *p* < 0.001) increased in MPN patients compared to matched controls (Malin Hultcrantz et al., 2014). While this application recognizes the various MPN manifestations that could contribute to thrombosis, such as leukocytosis or inflammation, several lines of evidence suggest enhanced platelet activation is involved in pathogenesis of thrombosis in MPN (Marin Oyarzun and Heller, 2019; Matsuura et al., 2020). For instance, platelet surface P-selectin (CD62P) is elevated in ET and PV patients, and correlates with history of thrombosis (Falanga et al., 2005; Alvarez-Larran et al., 2008). Increased levels of soluble forms of P-selectin and CD40L in ET patients also correlates with occurrence of thrombosis (Musolino et al., 1999; Viallard et al., 2002; Arellano-Rodrigo et al., 2009).

#### MPN and thromboembolism

It is well established that MPN increases the risk of venous thromboembolism [reviewed by Barbui et al. (2013)]. The incidence appears to be the highest in PV, with lower rates observed in ET and PMF (Barbui et al., 2010; Tefferi and Barbui, 2020). The JAK2^V617F^ mutation is correlated with thrombotic risk, which likely partially explains the higher rate seen in PV as 90%–95% of patients carry the JAK2^V617F^ mutation (Carobbio et al., 2011). Cardiovascular mortality was the main contributor to death in the largest observational study on PV, the European Collaboration on Low-dose Aspirin (ECLAP) study (Marchioli et al., 2005). Next to VTE, arterial thrombosis, coronary artery disease and congestive heart failure accounted for ∼41% of all deaths in this study. In an international collaborative study, the thrombohemorrhagic phenotype of patients with ET was evaluated; after a median follow-up of 6.2 years, 12% of the patients developed thrombosis (Carobbio et al., 2011). Interestingly, the rates of arterial thrombosis were increased 2-3 fold, compared to venous thromboembolic events. In a large European study following >700 patients with MF, the cumulative amount of major vascular events was found to be 7.2%, equating to 1.75 events per 100 patient years (Barbui et al., 2010). The majority of deaths in the study were due to leukemic transformation, infections and transplantation-related causes. Fatal cases due to cardiovascular events represent 0.39 deaths per 100-patient years and included a high rate of venous thrombosis related causes (including pulmonary embolism, portal vein thrombosis and Budd-Chiari syndrome).

The hallmark of thrombotic prevention in patients with PV/ET is the use of the antiplatelet agent aspirin. Aspirin has proven to be effective for primary prevention in PV (Landolfi et al., 2004). The data in ET is extrapolated from studies with PV who also have thrombocytosis and retrospective studies in ET patients (Landolfi et al., 2004). In the landmark trial studying PV patients, 3 year follow-up revealed a significant reduction in the composite primary endpoint of cardiovascular death, nonfatal myocardial infarction, major venous thromboembolism and stroke (Landolfi et al., 2004). Retrospective data from patients with ET revealed that antiplatelet therapy reduces the risk of VTE in patients harboring the JAK2^V617F^ mutation (Finazzi et al., 2012). In PMF however, the role of antiplatelet therapy is less clear and antiplatelet therapy is not recommended in unselected patients with PMF for primary prevention.

Outside of MPNs, anti-platelet agents are not currently routinely used to prevent VTE/ATE in patients with solid malignancies (Tao et al., 2021). The United States Preventative Services Task Force previously recommended anti-platelet agents for colorectal cancer prevention in patients aged 50–59, but this recommendation has been withdrawn due to a lack of evidence from an updated systematic review (US Preventive Services Task Force Davidson et al., 2022). The evidence in myeloproliferative neoplasms suggests that a more selective approach to anti-platelet therapy may be more effective in cancer and thrombosis prevention, rather than treating all-comers.

#### MPN and hemorrhagic complications

Patients with PV/ET are at risk of developing extreme thrombocytosis (defined as a platelet count >1,000,000/microL). No correlation has been found between the extent of thrombocytosis and the thrombotic risk of patients with MPN (Campbell et al., 2012). Rather, extreme thrombocytosis has been associated with a hemorrhagic phenotype. This is commonly attributed to an acquired von Willebrand syndrome (aVWS) (Etheridge et al., 2014). High molecular weight von Willebrand multimers are thought to bind to GPIb leading to accelerated proteolysis by a disintegrin and metalloproteinase with thrombospondin motifs-13 (ADAMTS13). Others have proposed that atypical cleavage of vWF multimers is due to increased baseline platelet and leukocyte activation. Alternatively, in a mouse model of endothelial cell specific expression of JAK2^V617F^, a similar defect in high molecular weight vWF multimers was observed (Etheridge et al., 2014). This observation postulates the question if vWF processing or secretion may be involved as well in the aVWS observed in patients with MPN. Ultimately, the clinical phenotype in patient is one of hemorrhage due to accelerated removal of high molecular weight vWF. The human phenotype is, however, heterogeneous. For example, analysis from the German Study Group for MPN revealed that in JAK2^V617F^ mutated ET >30% of the patients had suffered a vascular event, which included both arterial and venous events (Kaifie et al., 2016). Interestingly, major bleeding events were also observed in ∼8% of all patients.

### MPN-platelet communication leading to thrombosis

#### Extracellular vesicles

Once platelets are activated and primed by the tumor, there is increasing evidence that a procoagulant pool of platelets and platelet-derived EVs develop (Hua et al., 2015). This procoagulant pool consists of phosphatidylserine-positive (PS+) platelets. In patients with several cancers, including colon cancer and non-small cell lung cancer PS+ platelets were found to be elevated (Zhao et al., 2016; Ma et al., 2017). Similarly, PS+ platelet-derived EVs (defined as bearing CD41 and CD42) are elevated in gastric cancer, breast cancer and several gastrointestinal tumors (Alvarez et al., 2022; Contursi et al., 2023). Similar to the case of solid tumors, PV or ET manifests with increased PS+ platelet subsets (Presseizen et al., 2002; Tan et al., 2013; Ji et al., 2020). A prior history of thrombosis in patients with MPN was correlated with raised EV levels compared to MPN patients without thrombosis (Zhang et al., 2017). Interestingly, in patients with MPN, platelet dependent thrombin generation is markedly elevated (Panova-Noeva et al., 2013). These studies postulate that procoagulant platelets may be causative for the thrombotic tendency in patients with MPN. Compared to healthy controls, patients with PV or ET were found to have increased resistance to thrombomodulin using a thrombin generation test. Size exclusion suggested involvement of microparticles <0.22 μm in this observation, as filtration increased the sensitivity to thrombomodulin (Duchemin et al., 2010). More recently, PV patients were found to have an increased level of platelet derived EVs. Proteomic evaluation of platelet derived EVs in MPN patients revealed upregulation of proteins related to platelet activation, immune and inflammatory response and angiogenesis (Fel et al., 2019). Overall, these findings point towards an important role for EVs in the thrombotic phenotype of MPN patients. Likely, other vascular cells contribute to the EV pool; the relative contributions of different EVs to the thrombotic phenotype need to be further established. For example, endothelial cells harboring the JAK2^V617F^ have increased apoptosis and generate tissue factor positive microparticles.

#### Platelet dysfunction

After identification of the gain-of-function mutation in the JAK2 kinase, JAK2^V617F^, several groups dedicated their efforts to develop mouse models that allow for understanding of the effect on intrinsic hematopoiesis and PV/ET progression to myelofibrosis or secondary leukemia (Baxter et al., 2005; James et al., 2005; Jones et al., 2005; Kralovics et al., 2005; Levine et al., 2005). Initially, retroviral approaches were used to overexpress JAK2^V617F^, which led to a successful myeloproliferative phenotype with hallmarks of PV, including splenomegaly, erythrocytosis and altered hematopoiesis. While increased numbers of abnormal megakaryocytes were observed in all models, only models with low-expressing JAK2^V617F^ were found to have a thrombocytosis. The authors postulated that low levels of JAK2 kinase activity skews towards a more megakaryocytic phenotype, whereas high levels would lead to a PV-like phenotype (Tiedt et al., 2008). Another hypothesis that could explain heterogeneity between patients with ET and PV is that a secondary acquired mutation in addition to the JAK2^V617F^ would allow for the evolution to different phenotypes within the MPN spectrum.

To further characterize constitutive activation of the JAK2 kinase, both transgenic and knock-in models were developed (Morgan and Gilliland, 2008; Xing et al., 2008; Li et al., 2010; Mullally et al., 2010). While these models have extensively furthered our understanding of the role of JAK2^V617F^ in altering the hematopoietic niche and signaling pathways that are involved in disease progression, there is less data on whether these models reproduce the abnormal thrombotic phenotype as seen in humans with MPN. In the next paragraphs, we will summarize observations from studying thrombohemorrhagic complications and the role of platelets in mice harboring the JAK2^V617F^ mutation.

Using the Li et al. JAK2^V617F^ knock-in model (*human JAK2* ^*V617F*^ *, Mx1-Cre-promoter*), which resembles an ET-like phenotype, megakaryocytes and platelets were characterized to assess their role in bleeding and clotting (Hobbs et al., 2013). Higher ploidy (>8n) was observed in the JAK2^V617F^ bone marrow of these mice, indicating increased MK maturation reminiscent of patients with ET. Furthermore, JAK2^V617F^ MKs showed increased proplatelet formation and increased migration towards the vascular niche in a model using fibronectin-coated Dunn chambers. JAK2^V617F^ platelets were hyperactive to several agonists, including activators of GPVI, PAR1 and P2Y12-receptors. These findings translated into decreased tail vein bleeding after tail-clipping and increased platelet aggregates using an *in vitro* flow chamber model of shear.

In a model of secondary MF, using the JAK2^V617F^ transgenic model as described by Xing et al. (*human JAK2* ^*V617F*^ *, vav-Cre-promoter*) aged mice (∼30 weeks) were used to evaluate its thrombogenic phenotype (Matsuura et al., 2020). Earlier in their life, these mice have hallmarks of PV, but upon aging they develop marked splenomegaly, normalization of their erythrocytosis and the development of increased reticulin in their bone marrow and spleens, consistent with secondary MF. In models of thrombosis, platelet thrombi were shown to be unstable with rapid embolization after injury; a state known as typical inducer of small vessel occlusion. Similarly, time to occlusion after FeCl_3_ carotid artery injury or tail vein clipping was prolonged. Platelet response to collagen was diminished in aggregation, with decreased dense granule secretion of ATP/ADP. Using electron microscopy, it was shown that platelets of these mice showed a dense granule deficiency. Intriguingly, dense granule dysregulation has been observed in patients with PV/ET as evidenced by a decrease in mepacrine uptake (Caranobe et al., 1984).

#### The role of NETs

A different group assessed the JAK2^V617F^ knock-in model as developed by Mullaly et al. (*mouse JAK2* ^*V617F*^, *E2a-Cre-promoter*) (Wolach et al., 2018). This model leads to a PV-like disorder, which ultimately evolves into MF. The platelet phenotype of these mice represents a mild GPVI deficiency, hyporesponsive platelets to agonists targeting PAR1 and GPVI and impaired thrombus formation using a FeCl_3_ induced injury model. Tail vein bleeding was found to be prolonged compared to littermate controls. These findings were confirmed in a separate investigation. In this study the role of platelet-neutrophil interactions was examined. The JAK2^V617F^ model as developed by the Mullaly group displays increased neutrophil extracellular traps (NETs) in thrombosis models known to be dependent on NETs, such as the IVC ligation model. Interestingly, the thrombotic phenotype of these mice could be blunted by administration of the JAK2 inhibitor ruxolitinib.

In a different study, the sulfhydryl containing compound N-acetylcysteine (NAC), was found to increase lifespan of JAK2^V617F^ knock-in mice (Craver et al., 2020). The exact mechanism by which NAC exerts its effect is likely multifactorial as it is an antioxidant, mucolytic and has anti-inflammatory properties. Interestingly, JAK2^V617F^ knock-in mice treated with NAC displayed diminished pulmonary thrombus formation. JAK2^V617F^ knock-in platelets increased NETosis and pre-treatment with NAC diminished both platelet-leukocyte interactions and subsequent NETosis.

Neutrophils from patients with MPN have been evaluated to assess if they contribute to the prothrombotic state. Two independent studies found that unstimulated neutrophils from MPN patients did not increase NET formation (Marin Oyarzun et al., 2016; Wolach et al., 2018). A separate study, however, observed the opposite (Guy et al., 2019). The apparent contradictory effects may be explained by differences in cytoreductive therapy and the small sample size in the first two studies. The latter study selected patients who had prior thrombosis. Wolach et al. found that JAK inhibition in both healthy control and MPN neutrophils prior to stimulation with a calcium ionophore or phorbol myristate acetate (PMA) led to diminished NET formation (Wolach et al., 2018). Overall, these observations raise the potential for JAK-STAT inhibition leading to decreased NET formation and ultimately reducing venous thrombosis.

### Platelet biomarkers of thrombosis in MPN

Risk scoring systems, such as the International Prognostic Score for Essential Thrombocythemia (IPSET-thrombosis) were derived from a cohort of 891 subjects with ET as defined by the WHO (Barbui et al., 2012). This score compromises four different risk factors, including age >60, the presence of cardiovascular risk factors (such as hypertension, diabetes, active tobacco use), a history of thrombosis and the presence of the JAK2^V617F^ mutation. This stratifies patients into three risk groups (high-risk, intermediate risk and low risk of thrombosis). Other markers, such as leukocytosis, have also been found to independently predict thrombosis in ET (Carobbio et al., 2019). However, it remains currently unclear how to incorporate this into the current scoring systems. Better and more personalized markers of thrombotic risk are warranted to assist physicians and patients to select the appropriate thromboprophylaxis strategy in the case of MPNs.

An increased mean platelet volume (MPV) is a known predictor of arterial thrombosis (Noris et al., 2016). Larger platelets show an increased propensity for aggregation *in vitro*. In several retrospective studies, however, there was no difference in MPV in patients with MPN with or without thrombosis (Falanga et al., 2007). Several non-platelet hemostatic markers have been shown to be elevated in patients with MPN. Leukocyte activation markers, including elastase, CD11b, CD14, LAP and CD40L were all shown to be upregulated in MPN (Falanga et al., 2007). Protein disulfide isomerase (PDI), an endoplasmic reticulum resident critical for ensuring proper protein folding, has been shown to play an important role in the pathogenesis of thrombosis (Cho et al., 2008; Jasuja et al., 2012; Bekendam et al., 2016). PDI is shown to be released by platelets, endothelial cells and neutrophils upon activation (Essex et al., 1995; Essex and Li, 1999; Jasuja et al., 2010; Hahm et al., 2013). Plasma levels of PDI were found to be elevated in patients with JAK2^V617F^ mutation and represented an independent biomarker of thrombosis (Sharda et al., 2021). Interestingly, in this cohort, classical markers of increased thrombotic risk, including leukocytosis and age were not correlated with PDI levels. Further studies are warranted to assess if PDI acts as a functional effector or can be used as a biomarker. Interestingly, a recent trial using isoquercetin, a known inhibitor of PDI, reduced D-dimer levels and soluble P-selectin in patients with advanced malignancy (Zwicker et al., 2019). Similarly, exploratory data showed that platelet dependent thrombin generation was PDI dependent and inhibited in cancer patients who received isoquercetin (Stopa et al., 2017).

RNA sequencing of purified platelets in patients with MPN has revealed distinct platelet signatures have been identified. IL1RAP, a subunit of the IL-1 type 1 receptor, was transcriptionally upregulated in platelets of patients with PV or ET who had had thrombosis compared to patients without thrombosis (Gangaraju et al., 2020). Another study evaluating platelet transcriptome profiles, identified a strong thromboinflammatory profile in samples from patients with ET and PV (Shen et al., 2021). Several interferon inducible genes (e.g., IFITM2, IFITM3, IFITM10), interleukin receptor proteins (IRAK1, IL15) and coagulation factor V were identified as potential mediators of the thrombotic phenotype in patients with MPN. Further validation of these identified markers as biomarkers will be prudent for future therapeutic evaluation, furthering of our understanding of the thrombotic pathophysiology and for refining and application into risk prediction models. One limitation of current RNA transcriptomics in platelets is the difficulty to perform this reliable on a single cell level, low yields of RNA remain a substantial limitation (De Wispelaere and Freson, 2022).

In recent years, it has become clear that megakaryocytes and megakaryocyte progenitors are heterogeneous. In a seminal paper by Sun et al. (2021) it was described that MKs consist of four different clusters. Cluster 1 is comprised of cells in all ploidy stages, with high expression of genes related to DNA replication and cell cycle progression. This cluster was classified as active cycling MKs. Cells in cluster 2, expressed regulatory HSC markers, such as Pf4 and Igf1. Most of the MKs were high in ploidy (≥8n) and are classified as HSC-niche supporting. Cells in cluster 3, consisted mainly of lower ploidy cells (≤8n) and expressed an inflammatory response signature (Lsp1, CXCR4 and CD53). Cells in cluster 3 are termed immunoregulatory. Cells in cluster 4 express key markers involved in platelet production and hemostasis, such as vWF, GPIba, Gp5 and Gp6. As platelet production correlates with higher ploidy MKs, MKs in this cluster had higher numbers of high ploidy cells. This change in paradigm has altered our thinking of how to view both MKs and their platelet offspring. It will be of interest to see if identification of subtypes of platelets either through single RNA sequencing or measurement of cell surface markers will lead to a better understanding of the role of platelets in the thrombotic phenotype of MPNs.

Finally, there is increased tendency for NETosis in MPN, (Marin Oyarzun et al., 2016), however, it is not clear if these NETs can serve as biomarkers, and further studies are needed to assess if NETosis is the main driver of the thrombotic phenotype in MPN or if it plays a facilitating role.

## Conclusion

**FIGURE 1:**
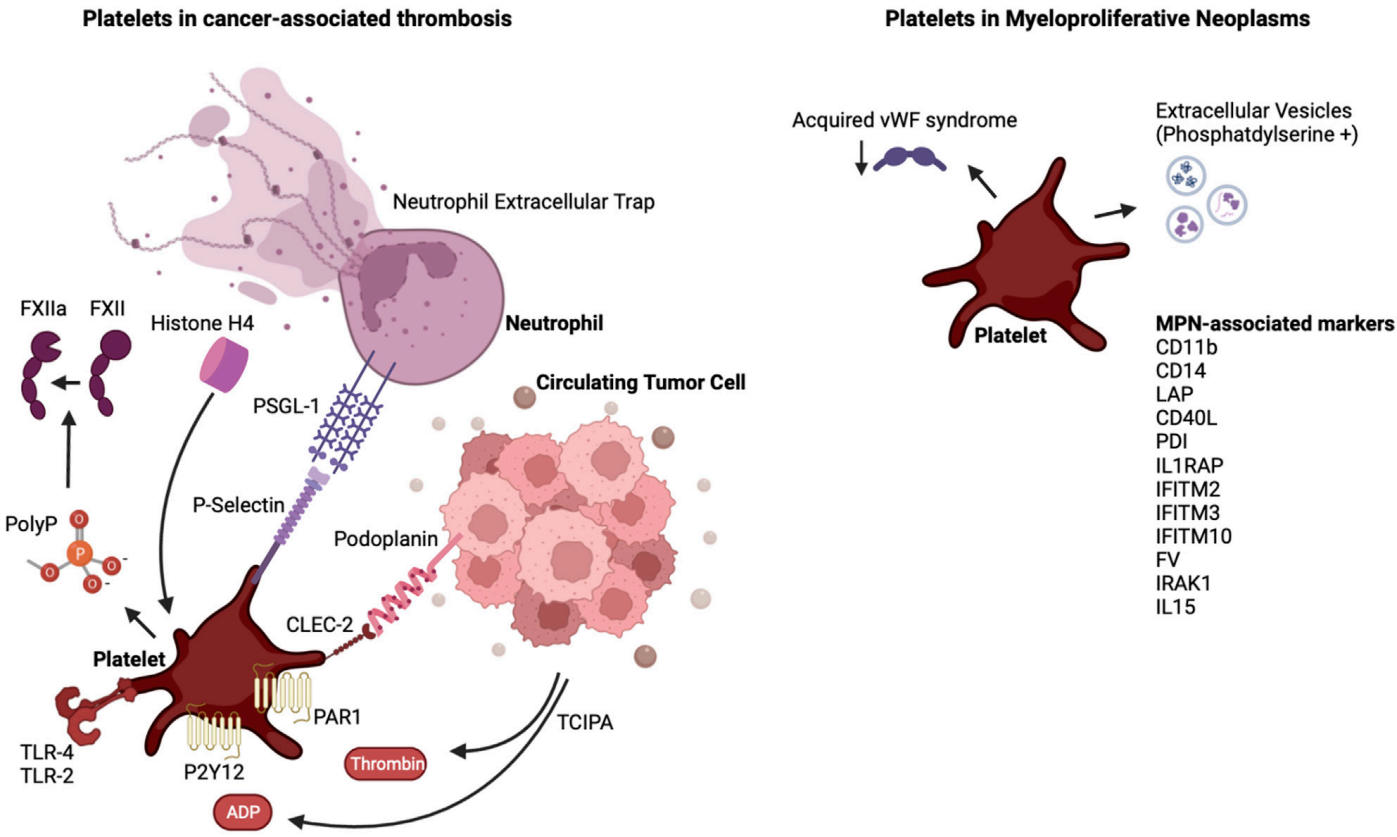
The interplay of platelets and cancer and the case of MPN. Shown are platelet-activating factors released by tumor cells, and the link of these tumor educated platelets to neutrophils. In the case of MPN, denoted are factors released or expressed on the surface of MPN cells and in the plasma of MPN patients which, as reviewed in the text, influence platelet and extracellular vesicles properties. Abbreviations are as denoted in the text.

Thrombosis remains a significant cause of morbidity and mortality in patients with cancer, especially in those with MPN. Platelets possess a versatile machinery that allows them to interact with tumor cells, vascular cells and coagulation proteins. Mouse models of MPN have advanced our understanding of natural progression of MPNs and have led to increased understanding of thrombotic mediators in mice harboring the JAK2^V617F^ mutation. It will be prudent to evaluate the thrombotic phenotype in mice harboring the known CALR type 1 and 2 mutations or the MPL mutation, which have been developed in recent years. The current experimental models address MPN as a monogenic disease, and as such do not address the genetic complexity of MPN in humans. The absence of spontaneous splanchnic vein thrombosis in mouse models is different from the human MPN where this can be a pathognomonic finding. JAK inhibition with ruxolitinib reverses the myeloproliferative phenotype in mice, but the benefit for altering disease process leading to overall survival in humans remains controversial. Similarly, the role of JAK inhibition in the prevention of thrombosis remains unclear, although a recent phase II trial with ruxolitinib showed a benefit in thromboembolic event-free survival. Crizanlizumab, a monoclonal antibody targeting the interaction between PSGL-1 and P-selectin, is currently being evaluated in myelofibrosis (NCT04097821). The role of the podoplanin/CLEC-2 axis has not been evaluated in the setting of MPN. As more evidence arises about the heterogeneity within the platelet pool, future studies could evaluate how platelets of different sizes or age are influenced by and are influencing tumors. Ultimately, this will lead to better targeted anti-platelet agents and biomarkers to help minimize the prevalence of CAT and to better risk stratification. Figure 1.
